# Anti-*Candida* Antibodies of Patients with Invasive Candidiasis Inhibit Growth, Alter Cell Wall Structure, and Kill *Candida albicans* In Vitro

**DOI:** 10.1007/s11046-023-00819-w

**Published:** 2024-02-07

**Authors:** Giulia Carrano, Inés Arrieta-Aguirre, Ander Díez, Marta Bregón-Villahoz, Iñigo Fernandez-de-Larrinoa, María-Dolores Moragues

**Affiliations:** 1 Department of Immunology, Microbiology and Parasitology, Faculty of Pharmacy, University of the Basque Country UPV/EHU, Vitoria-Gasteiz, Araba Spain; 2 Department of Nursing I, Faculty of Medicine and Nursing, University of the Basque Country UPV/EHU, Leioa, Bizkaia Spain; 3 Department of Applied Chemistry, Faculty of Chemistry, University of the Basque Country UPV/EHU, Donostia-San Sebastian, Gipuzkoa Spain

**Keywords:** *Candida albicans*, Invasive candidiasis, Antibodies, Growth inhibitor, Fungicides

## Abstract

Invasive candidiasis (IC), caused by *Candida* yeasts, particularly *Candida albicans*, poses a significant threat with high mortality rates. Diagnosis is challenging due to *Candida*'s common presence in human microbiota. To address this, our research group developed an immunofluorescence assay detecting *Candida albicans* Germ Tube Antibodies (CAGTA) in IC patients. CAGTA, indicative of invasive processes, is associated with a lower mortality rate in ICU patients. Based on this premise, this study aims to provide results regarding the lack of knowledge about the potential activity of CAGTA against invasive infections in humans caused by the fungus *Candida albicans*. Therefore, in order to characterize the activity of CAGTA produced by patients with IC, we used sera from 29 patients with IC caused by either *C. albicans* or non-*albicans Candida* species. Whole serum IgG antibodies were fractionated into anti-blastospores, CAGTA-enriched, and purified CAGTA and the assessments included XTT colorimetric assays for metabolic activity, CFU counts for viability, and microscopy for growth, viability, and morphological analysis. The CAGTA-enriched IgG fraction significantly reduced the metabolic activity and viability of *C. albicans* compared to anti-blastospores. Purified CAGTA altered germ tube cell wall surfaces, as revealed by electron microscopy, and exhibited fungicidal properties by DiBAC fluorescent staining. In conclusion, antibodies in response to invasive candidiasis have antifungal activity against *Candida albicans*, influencing metabolic activity, viability, and cell wall structure, leading to cell death. These findings suggest the potential utility of CAGTA as diagnostic markers and support the possibility of developing immunization protocols against *Candida* infections.

## Introduction

Invasive Candidiasis (IC) is a fungal infection caused by yeasts of the genus *Candida*, among which *Candida albicans* is the most frequently isolated, affecting more than 250,000 people worldwide every year and cause more than 50,000 deaths [1]. Some species of *Candida* are common colonizers of the skin, and of the gastrointestinal, genitourinary and respiratory tract mucous membranes. Nevertheless, under certain circumstances, they can cause both superficial and invasive infections, and the latter are associated with high mortality rates due to the diagnostic difficulties derived from the absence of pathognomonic signs [2].

Despite some improvement in diagnostic methods and the introduction of new drugs for treatment, IC is still a major problem in critically ill patients [3–5]. Other risk conditions for an invasive fungal disease (IFD) are immunosuppression, HIV infected patients, recipients of hematopoietic or solid organ transplantation, and long stay in the ICU [6, 7].

Globally, the incidence of *C. albicans* isolates is decreasing (65.3% to 48.4%), in favor of non-*albicans Candida* species (NAC) such as *Candida glabrata* (from 11.3 to 18.2%), *Candida parapsilosis* (6% to 17.1%) or *Candida tropicalis* (7.2% to 10. 6%) [8]. In Spain, nowadays, *C. albicans* is still the major cause of IC (47.7%), followed by *C. parapsilosis* (22%), *C. glabrata* (15.8%), *C. tropicalis* (9.3%) and *Candida krusei* (2.8%) [9].

Blood culture remains the “gold standard” for diagnosis of IC or, alternatively, histopathological evidence in normally sterile sites, but both techniques have low sensitivity, and a long turnaround time (72–96 h) leading to a delay in specific treatment that results in increased mortality [10]. In this line, a multicenter retrospective study conducted in 23 ICUs of 9 European countries reported an IC incidence of 7.07 episodes per 1000 admissions with a 30-day mortality of 42% [11].

In an attempt to improve the diagnostic resources for IC, our group of research developed an indirect immunofluorescence (IIF) assay detecting antibodies that recognize specific antigens located on the cell wall surface of *C. albicans* germ tubes; such antibodies are associated to the invasive candidiasis process and were termed CAGTA (*Candida albicans* Germ Tube Antibodies) [12–18]. The detection of CAGTA has achieved very interesting results, even in neutropenic patients, and its diagnostic value has been proved successful for monitoring antifungal therapies [19]. A Spanish multicenter study with 53 IC patients admitted to ICU evidenced that those with increased CAGTA titers along with antifungal treatment greatly reduced their associated mortality rate from 61.2 to 22.7% [20]. This aspect is related to an efficient anti-*Candida* response that requires the cooperation of different mechanisms of the immune system [21–24]. In this regard, antibodies confer protection against fungal infections by opsonization and phagocytosis, complement activation and antibody-dependent cell toxicity [25, 26]. In addition to direct neutralization of fungi and their antigens, antibodies can inhibit fungal growth, modify gene expression, signaling and lipid metabolism, induce iron starvation, and reduce polysaccharide release and biofilm formation [6, 24].

Furthermore, the identification of specific antigens that are recognized by CAGTA, such as hyphal wall protein 1 (Hwp1), agglutinin-like sequence protein 3 (Als3), and methionine synthase protein 6 (Met6) has served to develop experimental immunoassays to detect antibodies that may help to the diagnosis of IC in patients at risk, even in immunocompromised ones [27]. In this line, since CAGTA have been related to a better prognosis of the infection, members of our group have assayed some epitopes of the antigens recognized by CAGTA as a complex peptide vaccine conjugated to KLH (3P-KLH) for protection in a murine model of hematogenously disseminated candidiasis, with promising results (Diez et al. data not published). In fact, patients who survived systemic infections developed strong antibody responses to certain *C. albicans* proteins that could help as a prognostic signature for an IC clinical-outcome prediction model [28–30].

An alternative approach to the management of IC is the development of prophylactic treatments based on monoclonal or polyclonal antibodies [31].

Therefore, the aim of this study is to assess the efficacy of antibodies raised by patients with Invasive Candidiasis against *Candida albicans*, with a particular focus on CAGTA. The investigation involves recording the effect of these antibodies in vitro on the metabolic activity and viability of the fungus, as well as on the structure of its cell wall. This research is essential for gaining insights into the immune response to invasive Candidiasis, potentially contributing to the development of therapeutic strategies or vaccines.

## Material and Methods

### Yeast Strain and Culture Conditions

*Candida albicans* SC 5314 (Stanford DNA Sequencing and Technology Center, Stanford, USA) was used in all experiments. Yeast cells were grown on Sabouraud Dextrose Agar (SDA; Difco, Sparks, MD, USA) at 37 °C for 48 h. To obtain germ tubes, cells grown in SDA were inoculated into TC199 medium (Sigma-Aldrich, St. Louis, MO, USA) and incubated at 24 °C overnight; blastospores were collected by centrifugation and inoculated into four volumes of TC199 medium (Sigma-Aldrich, St. Louis, MO, USA) preheated at 37 °C. The germ tubes were collected after 4 h of incubation at 37 °C and 120 rpm.

In order to prepare the inoculum for the experiments, yeasts were freshened overnight in SDA at 37 °C. Then, 2–3 colonies were inoculated into Sabouraud Dextrose broth (SDB, Difco, Sparks, MD, USA) and incubated overnight at 24ºC and 200 rpm. Yeast cells were collected by centrifugation at 2500 rpm for 5 min and washed three times with sterile PBS. The concentration of the inoculum was adjusted to 10^6^ cell/ml by using a Bürker chamber (Hirshmann, Germany) [32].

### Patients and Sera

Immune sera were obtained from 29 patients with IC from different clinical units (Hospital Severo Ochoa-Leganés-Madrid and University Hospital Cruces-Barakaldo-Bizkaia, Spain) (Table 1). Group I: 15 patients diagnosed with invasive *C. albicans* infection. Group II: 14 patients infected with NAC species.

**Table 1:** Characteristics of sera of 29 patients diagnosed with invasive candidiasis caused by different species of *Candida*

Group I: *Candida albicans* patients			Group II: non-*albicans Candida* spp. patients			
	Unit	CAGTA (reverse titer)^c^		Unit	CAGTA (reverse titer)^c^	Clinical isolate
1	Geriatric	5120	16	ICU^a^	2560	*C. glabrata*
2	ICU^a^	2560	17	ICU^a^	320	*C. parapsilosis*
3	ICU^a^	1280	18	General surgery	160	*C. glabrata*
4	ICU^a^	1280	19	Hematology	160	*C. parapsilosis*
5	ICU^a^	1280	20	ICU^a^	160	*C. parapsilosis*
6	Internal medicine	1280	21	ICU^a^	160	*C. parapsilosis*
7	REA-ICU^b^	320	22	ICU^a^	160	*C. parapsilosis*
8	Internal medicine	160	23	Internal medicine	160	*C. tropicalis*
9	Internal medicine	40	24	General surgery	80	*C. glabrata*
10	REA-ICU^b^	40	25	ICU^a^	20	*C. tropicalis*
11	General surgery	20	26	ICU^a^	10	*C. parapsilosis*
12	Hematology	20	27	Hematology	0	*C. parapsilosis*
13	Hematology	10	28	Hematology	0	*C. glabrata*
14	Hematology	10	29	Hematology	0	*C. glabrata* and *C. tropicalis*
15	Internal medicine	0				

^a^*ICU* Intensive Care Unit

^b^*REA-ICU* Reanimation and Intensive Care Unit

^c^*CAGTA*: reverse titer of antibodies against germ tubes of *C. albicans* assayed by indirect immunofluorescence. Serum dilution equal or greater than 1/160 was considered positive for IC

CAGTA titer of sera was assessed by indirect immunofluorescence as previously described [13], and based on their antibody titer they were classified as HIGH-titer sera (1/160–1/5120) or LOW-titer sera (0–1/80). The sera are included in an anonymized samples collection (C.0005025) registered by the Instituto de Salud Carlos III-Spain. This study was approved by of the Institutional Committee for Research with Human Beings (CEISH) of the Ethics Commission for Research and Teaching (CEID) of the University of the Basque Country (CEID) with reference number CEISH/216RM/2013/MORAGUES TOSANTOS.

### Serum IgG Fractions and Purification of Antibodies

Immune sera were fractionated to run the different assays as described previously [31]. Serum whole IgG class antibodies (w-IgG) were purified with Melon™ Gel IgG Spin Purification Kit (ThermoFisher Scientific, St. Louis, MO, USA). The CAGTA enriched fraction of serum IgG (CAGTA-enr IgG) was obtained after incubating w-IgG with an equal volume of heat-killed *C. albicans* blastospores (10^10^ cell/ml of PBS) for 2 h at room temperature, followed by centrifugation at 2500 rpm for 5 min to remove the blastospores. The anti-blastospore antibodies (antiBl-IgG) adsorbed onto the surface of the yeast cells were eluted by gentle shaking in 2.5 M sodium iodide (Sigma-Aldrich, St. Louis, MO, USA) at room temperature for 1 h, and blastospores were spun down. The eluted antiBl-IgG fraction was dialyzed (MWCO 12,000–14,000 Da; Medicell International, London, UK) against PBS and concentrated with polyethylene glycol 20,000 (Merck, Hohenbrunn, Germany).

**Fig. 1:**
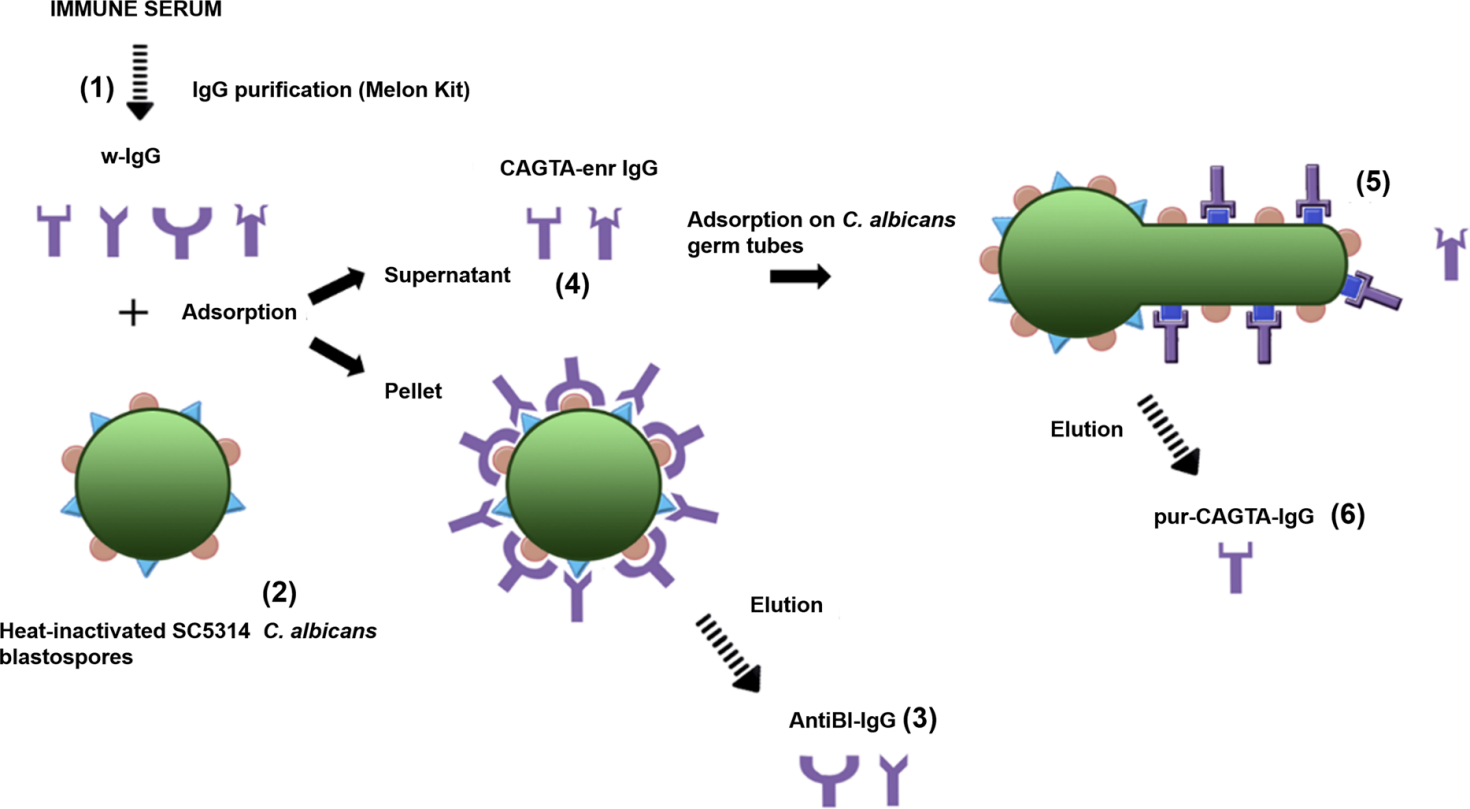
Scheme of the process for obtaining different serum fractions from patients with invasive candidiasis. Serum w-IgG from immune serum (1) was purified using the Melon kit. Then, it was incubated with heat-killed *C. albicans* yeast (2) to retain the antiBL-IgG (3) that were eluted after being centrifuged. The supernatant containing CAGTAs was termed "CAGTA enriched fraction" (CAGTA-enr IgG) (4). The pur-CAGTA (6) were eluted from the surface of *C. albicans* germ tubes that were incubated with the CAGTA-enr fraction (5). Abbreviations: whole IgG (w-IgG), anti-blastospores antibodies (AntiBL-IgG), CAGTA enriched fraction (CAGTA-enr IgG), and purified CAGTA (pur-CAGTA)

Purified CAGTA (pur-CAGTA) were eluted from *C. albicans* germ tubes after being incubated with an equal volume of CAGTA-enr IgG fraction for 1 h at room temperature with gentle agitation. Purified CAGTA were eluted, dialyzed and concentrated following the same protocol described for antiBl-IgG. The scheme of the serum fractionation process is depicted in Fig. 1.

The protein concentration of serum IgG fractions was estimated with Pierce™ Coomassie Plus (Bradford) Assay Kit (ThermoFisher Scientific, St. Louis, MO, USA) according to the manufacturer's instructions.

### Evaluation of Metabolic Activity of *Candida albicans*

The effect of the antibodies on *C. albicans* was evaluated by measuring the cell metabolic activity with the colorimetric XTT assay.

*Candida albicans* blastospores grown overnight in SDB at 30 °C and 120 rpm, were suspended in fresh medium 10^6^ cell/ml, and distributed in a U-bottom 96-well microplate (Cellstar, Greiner bio-one, USA), 25 μl per well. Each well was supplemented with 25 μl of SDB containing the correspondent IgG fraction to reach different final concentrations (50, 100 or 200 μg/ml). Plates were incubated at 37 °C for 2.5 h with gentle shaking, and then centrifuged at 2500 rpm, The supernatant was discarded, and the metabolic activity of cells was estimated with the 2.3-bis(2-methoxy-4-nitro-5-sulfophenyl)2H-tetrazolium-5-carboxanilide-inner salt (XTT; Sigma-Aldrich, St. Louis, MO, USA), according to the protocol of Henriques et al. [33]. Briefly, cell pellets in each well were supplemented with 90 μl of XTT 0.75 mg/ml in PBS and 10 μl of phenazine methasulfate (PMS; Sigma-Aldrich, St. Louis, MO, USA) 0.32 mg/ml in ultrapure water, both sterile filtered (0.45 µm pore-size filters; Minisart, Sartorius, Spain), and they were incubated at 37 °C for 2 h. Then, each well content was homogenized and the absorbance at 492 nm (Abs _492 nm_) was registered using a spectrophotometer (Microplate Autoreader Elx808, Bio-Tek Instruments). Two different controls were included in every experiment, one without antibodies, and a second one with an equivalent concentration of an irrelevant non-specific human IgG (irr-IgG; Sigma-Aldrich, St. Louis, MO, USA).

### Evaluation of Cell Viability

After being exposed to different IgG fractions, *C. albicans* cell viability was estimated by inoculating aliquots of duplicate wells on SDA plates, and counting colony forming units (CFU) grown at 37 °C for 48 h.

Alternatively, viability was assessed by staining with fluorochromes as described by Bowman et al. [34]. 5, (6)carboxyfluorescein Diacetate (CFDA; Molecular Probes, Eugene, OR, USA) dyes live cells while bis(1,3dibutylbarbituric acid) trimethine oxonol (DiBAC; Molecular Probes, Eugene, OR, USA) dyes death cells.

Briefly, *Candida albicans* blastospores grown overnight in SDB at 30 °C and 120 rpm, were suspended in fresh medium 10^6^ cell/ml, and distributed in a U-bottom 96-well microplate (Cellstar, Greiner bio-one, USA), 25 μl per well. Each well was supplemented with 25 μl of PBS containing pur-CAGTA to reach the final concentrations of 20 μg/ml. Then the cells were incubated for 2.5 h at 37 °C with gentle shaking. Then, the cells were centrifuged at 2500 rpm for 15 min and the pellet was split in parallel in 100 μl of CFDA and DiBAC solutions.

CFDA 50 μg/ml was prepared in MOPS 3 (0.1 M MOPS–50 mM citric acid at pH 3.0) from a stock solution of CFDA 5 mg/ml in dimethyl sulfoxide. Cells were incubated with the dye in the dark at 37 °C for 45 min with gentle shaking and then stored on ice until analysis. DiBAC 2 μg/ml in MOPS 7 (0.1 M MOPS at pH 7.0) was prepared from a 1 mg/ml stock solution in ethanol, and cells were stained at room temperature for 1 h in the dark, washed twice with MOPS 7 and stored on ice until analysis. Parallel phase-contrast and fluorescent images of the same field were taken using AZ100 Multizoom microscope (Nikon, Japan) and analyzed through Image Fiji software (Version 2.0, USA).

### Estimation of Growth and Cell Morphology by Optical Microscopy

Cell growth and morphology of *C. albicans* treated with CAGTA-enr IgG 6.25–200 µg/ml for 2.5 h at 37 °C were assessed by optical microscopy. Growth conditions of planktonic cells were the same as indicated for XTT and CFU assays. Two controls were run in every experiment, one without antibodies, and a second one with an equivalent concentration of a human irr-IgG. Images were obtained with an inverted microscope Olympus Fluoview FV500 (Olympus, Japan) and analyzed through Image Fiji software (Version 2.0, USA).

### Electron Microscopy

*Candida albicans* blastospores grown overnight in SDB at 30 °C and 120 rpm, were suspended in fresh medium 10^6^ cell/ml, and 50 μl per well were distributed in a U-bottom 24-well microplate (Iwaki^®^, Cell Biology, USA), previously filled with a nitrocellulose membrane (pore size 0,2 µm, Protran^®^, GE Healthcare, UK), fitting the well surface. Then, each well was supplemented with 50 μl of SDB containing pur-CAGTA fraction to reach the final concentrations of 20 μg/ml. Cells were grown for 2.5 h at 37 °C. Negative controls were run without CAGTA. Then, cells were gently washed by aspiration in Sorenson’s buffer [0.133 M Na_2_HPO_4_, 0.133 M KH_2_PO_4_ (4:1, v/v), pH 7.2], treated with fixing solution (2% glutaraldehyde in Sorenson’s buffer) at room temperature for 1 h, and washed three times with 6% sucrose in Sorenson’s buffer. Cells were dehydrated using graded ethanol solutions (50%, 70% and 100%) for 5 min each. Then membranes were washed twice for 5 min each with 5 ml hexamethyldisilazane (Electron Microscopy Sciences, Hatfield, PA, USA), that was removed by vacuum filtration. Membranes were left to dry in the air and processed for electron microscopy at the Service of Analytic and High Resolution Microscopy in Biomedicine (SGIker, University of the Basque Country UPV/EHU). Images were assembled through Image Fiji Software (Version 2.0, USA).

### Statistical Analysis

All assays were performed in duplicate and repeated three times. Statistical analysis was performed using GraphPad Prism Software (Version 6.00, Boston, Massachusetts, USA) for Windows. Significant differences were assesed using Kruskal–Wallis test. *P* values < 0.05 were considered statistically significant.

## Results

### Effect of Serum IgG Fractions from Patients with Invasive Candidiasis on *Candida albicans* Metabolic Activity and Viability

**Fig. 2:**
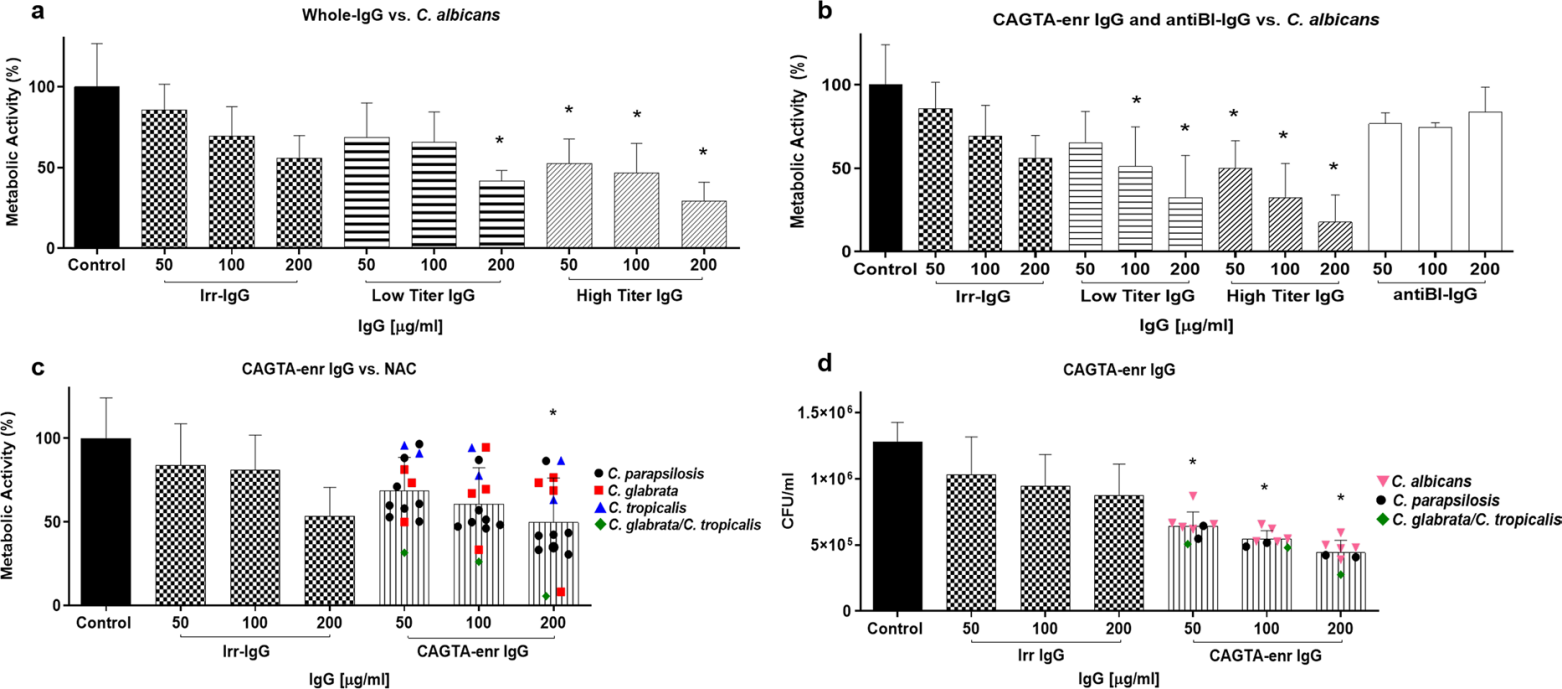
Metabolic activity and viability of planktonic cells of *C. albicans* SC5314 after being exposed to different human serum IgG fractions for 2.5 h in SDB at 37 °C. Control cultures were run without antibodies or with an Irr-IgG. Metabolic activity was estimated with XTT colorimetric assay (a, b and c), and viability was referred to as CFU/ml (d). Bars represent mean value ± SD from three independent experiments. *Statistical significance was established with reference to control cells using the Kruskal–Wallis test (*p* < 0.05). Abbreviations: whole IgG (w-IgG), CAGTA enriched fraction (CAGTA-enr IgG), anti-blastospores antibodies (AntiBL-IgG), irrelevant IgG (Irr-IgG) and NAC (non-*albicans Candida* spp).

Whole-IgG (w-IgG) from patients infected with *C. albicans* (group I) reduced the metabolic activity of *C. albicans* blastospores, as evaluated by the XTT assay, and the effect was concentration dependent; of note, the required amount of w-IgG that significantly reduced *C. albicans* metabolic activity was ≥ 50 µg/ml for immunoglobulins isolated from patients with high CAGTA titers (1/160–1/5120), whereas it was 200 µg/ml for low CAGTA titers (< 1/160) patients (Fig. 2a). Although the irrelevant commercial IgG (irr-IgG) showed a trend in reducing *C. albicans* metabolic activity compared to the control group, differences were not statistically significant.

The separate assay with CAGTA-enriched (CAGTA-enr IgG) and anti-blastospores IgG (antiBl-IgG) fractions revealed that the latter, up to 200 µg/ml, did not reduce the metabolic activity of *C. albicans*, relying the registered w-IgG inhibitory activity on the CAGTA-enr IgG fraction (Fig. 2b).

In addition, we also studied the effect of CAGTA-enr IgG fraction of sera from patients infected with non-*albicans Candida* spp. (NAC; group II). *Candida parapsilosis*, *C. glabrata* or *C. tropicalis* caused infections in 14 patients whose serum CAGTA titers were ≤ 1/320, except 1/2560 for one of the patients infected with *C. glabrata* (Table 1). As shown in Fig. 2c, the CAGTA-enr IgG fraction of NAC patients reduced *C. albicans* metabolic activity to a diverse extent, but their mean values were concentration dependent and resembled those of low-titer CAGTA patients infected with *C. albicans* (Fig. 2b).

CAGTA-enr IgG treatment of patients showing the highest effect on metabolic activity of *C. albicans* blastospores resulted in a significant reduction of CFU counts compared to untreated and irr-IgG treated control cells (Fig. 2d).

### Effect of Serum IgG Fractions from Patients with Invasive Candidiasis on *Candida albicans* Growth, Morphology and Viability

**Fig. 3:**
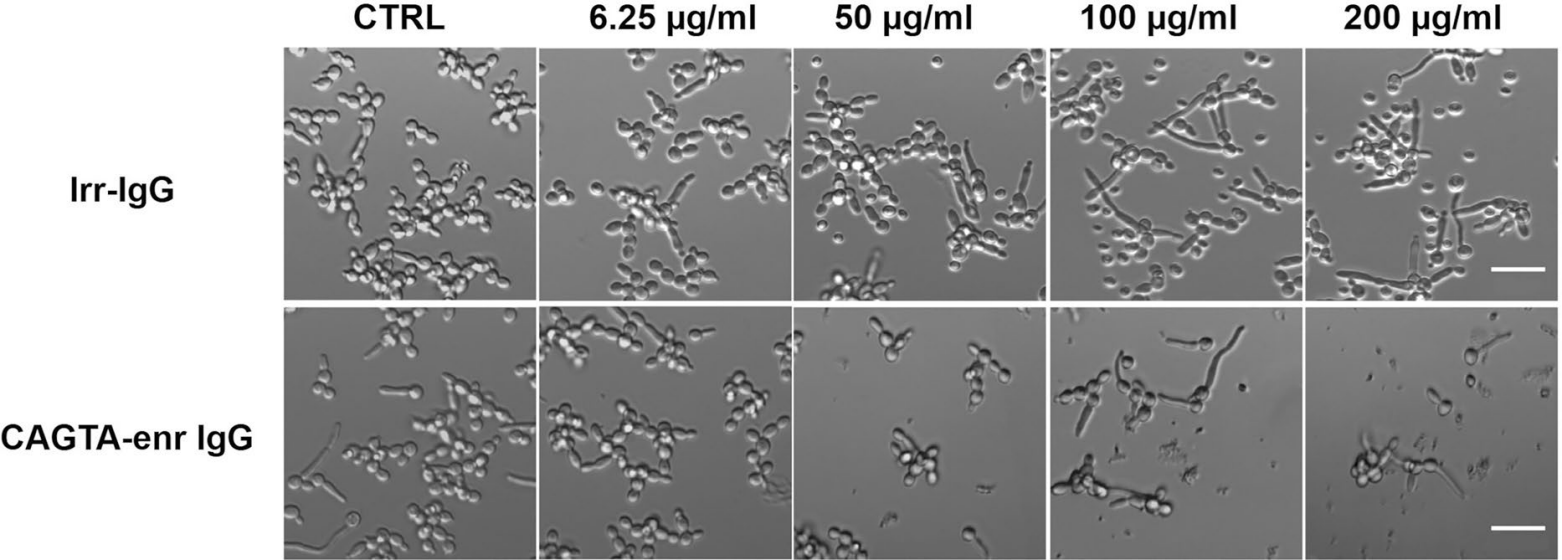
Phase contrast microscopy of *C. albicans* SC 5314 grown at 37 °C for 2.5 h in SBD supplemented with different concentrations of an Irr-IgG or CAGTA-enr IgG. CTRL were incubated without antibodies. Bar: 20 μm. Abbreviations: CAGTA enriched fraction (CAGTA-enr IgG), irrelevant IgG (Irr-IgG) and control cells (CTRL)

The optical microscopy images of *C. albicans* planktonic cells incubated with increasing concentrations of CAGTA-enr IgG fractions corroborated the above-mentioned findings (Fig. 3). While irr-IgG slightly reduced the growth of *Candida*, CAGTA-enr IgG reduced the visible yeast growth in a concentration dependent manner, though at the highest concentrations, we still could see some short and narrow germ tubes together with cell debris (Fig. 3).

**Fig. 4:**
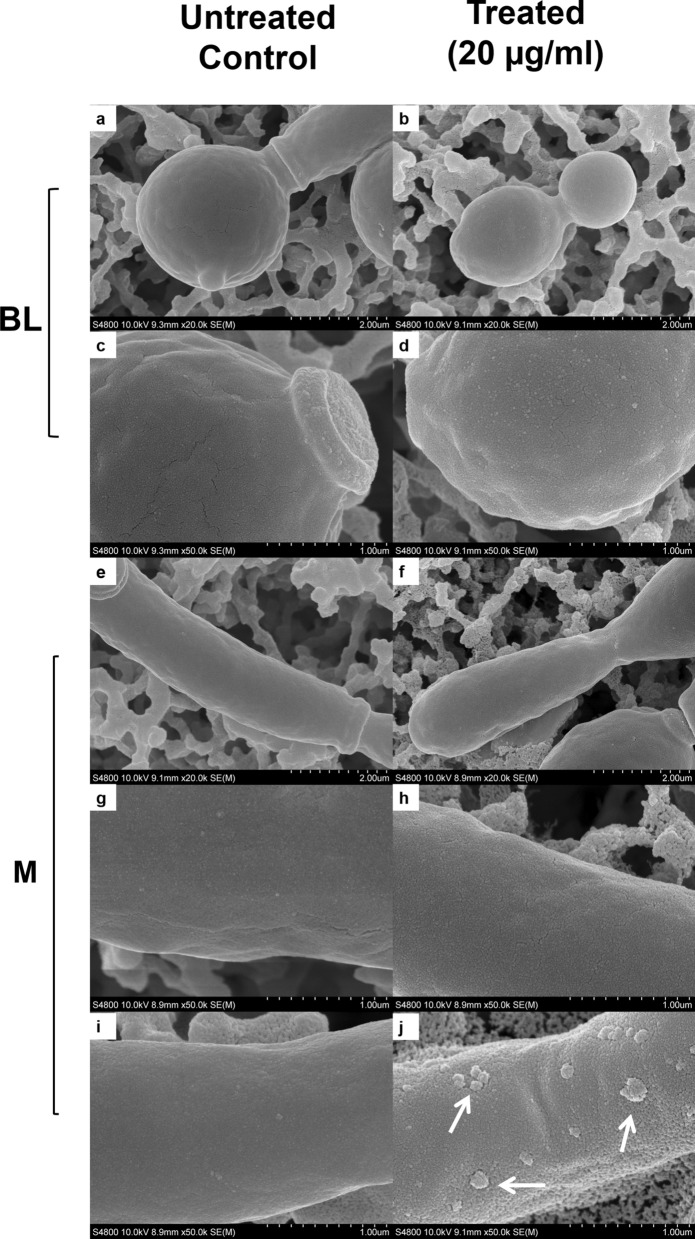
SEM images of *C. albicans* SC5314 blastospores (BL) and mycelia (M) grown at 37 °C for 2.5 h in SDB with pur-CAGTA 20 μg/ml (**b**, **d**, **f**, **h**, **j**) or without antibodies (**a**, **c**, **e**, **g**, **i**). Magnification: × 20,000 (**a**, **b**, **e**, **f**) and × 50,000 (**c**, **d**, **g**–**j**) for details. Arrows highlight surface protuberances. Abbreviations: blastospore (BL) and mycelia (M), purified CAGTA (pur-CAGTA)

**Fig. 5:**
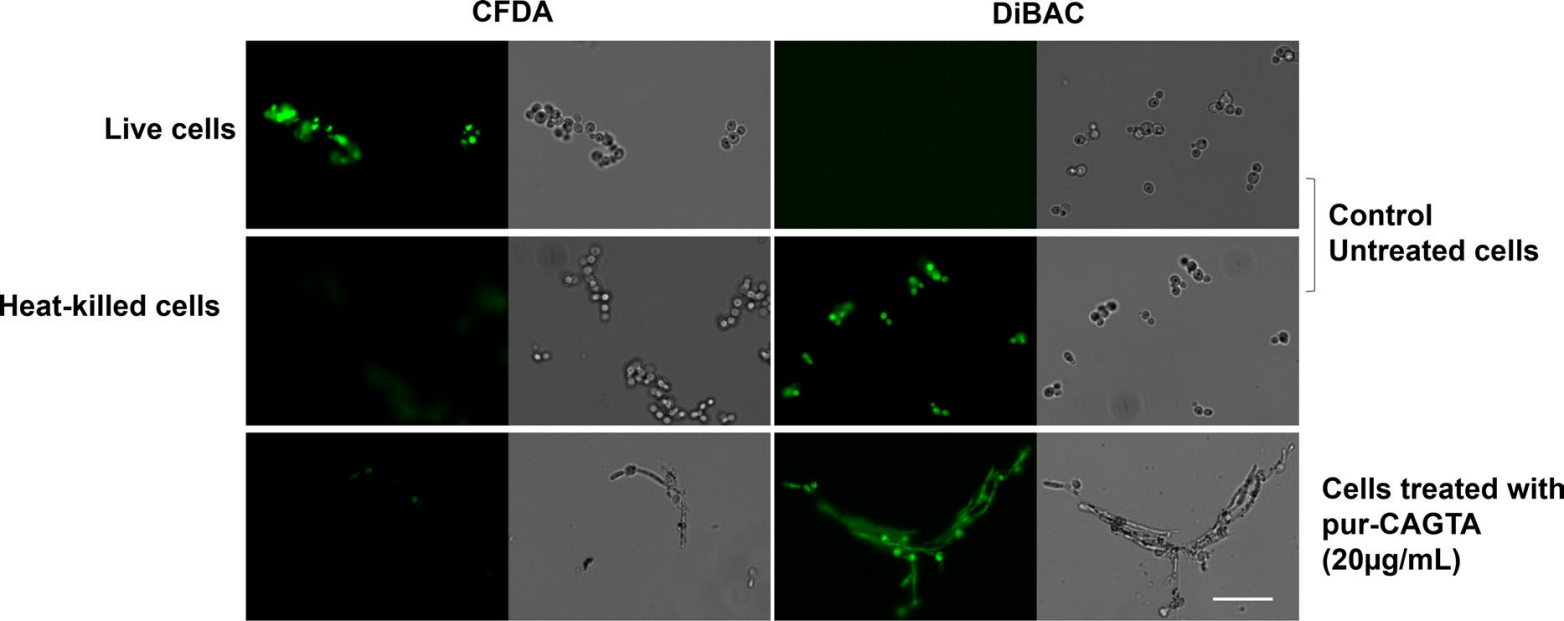
Photomicrographs of *C. albicans* SC5314 grown at 37 °C for 2.5 h in SBD with pur-CAGTA 20 μg/ml and then stained with the fluorescent dyes CFDA and DiBAC. Staining control consisted of cells grown in SBD without antibodies live and heat-killed. Paired images depict epifluorescent (on the left) and phase contrast microscopy (on the right) of the same field. Bar: 20 μm. Abbreviations: purified CAGTA (pur-CAGTA)

Consistent with optical microscopy findings, electron microscope images of pur-CAGTA IgG 20 µg/ml-treated *C. albicans* from patient #1—in group I (CAGTA titer 1/2560) revealed hyphae with altered morphology and surface fissures and protrusions (Fig. 4f, h, j), and surrounded by an altered matrix. On the contrary, the surface of untreated control yeast (Fig. 4a, c) and hyphae (Fig. 4e, g, i) as well as that of pur-CAGTA treated blastospores (Fig. 4: b and d) appeared regular and smooth. In addition, fluorescent DiBAC and CFDA staining of pur-CAGTA 20 µg/ml –treated *C. albicans* cells of the same patient #1, revealed a fungicidal effect for these antibodies (Fig. 5).

## Discussion

Invasive Candidiasis (IC) is a fungal infection caused by yeasts of the genus *Candida*, among which *Candida albicans* is the most frequently isolated. The opportunistic pathogen *C. albicans* has the ability to cause invasive infections, primarily attributed to its capacity to grow as hyphae. Various virulence factors associated with the cell wall are known to engage with host epithelial cells, initiating and fostering an immune response. Therefore, an effective anti-*Candida* response necessitates the collaboration of various mechanisms within the immune system, including the production of specific antibodies [21, 25, 26].

In addition to that, the development of the indirect immunofluorescence technique that detect CAGTA, conducted in our laboratory, has facilitated not only the diagnosis of IC in patients at risk, since it differentiates colonization from invasion [12, 13], but also, is reflects the importance of those antibodies in a better prognosis for patients with IC admitted at the ICU [20].

Therefore, in order to elucidate the importance of those antibodies, the present study was designed to investigate the antifungal activity of CAGTA against *C. albicans.*

Whole IgG antibodies from immune serum of patients infected with *C. albicans* reduced the metabolic activity and viability of *Candida* planktonic cells in vitro. These effects on the metabolic activity and/or viability of *C. albicans* have already been reported for antibodies developed in different animal models of invasive candidiasis, such as chickens [35], rabbits [31, 36], and Balb/C mice [37]. Furthermore, the monoclonal antibody C7 (mAb C7) derived from Balb/C mice immunized with *C. albicans*, exerted metabolic inhibition and fungicidal effect on this yeast [38] and, more recently, mAb Ca37 also inhibited the growth of *C. albicans *in vitro [39].

Interestingly, in human serum, the antiBl-IgG serum fraction showed slight effect on *C. albicans* planktonic cells and therefore, the CAGTA-enr fraction appeared as the main responsible for the observed inhibitory effect. Concomitantly, the microscopy images illustrated that CAGTA-enr IgG reduced the growth and filamentation of *C. albicans* yeast cells in a concentration dependent manner. Similarly, Uppuluri et al. demonstrated that anti-Als3 antibodies have the potential to disrupt various properties of *C. albicans* like adherence, filamentation, and biofilm formation [40]. Moreover, an Als3-specific monoclonal antibody (3D9.3) was observed to significantly decrease the adhesion of *C. albicans* germ tubes to human epithelial and vascular endothelial cells [41].

Electron microscopy images of *C. albicans* germ tubes exposed to CAGTA-enr human IgG revealed an altered cell wall surface with protrusions, whereas the blastospores retained their original smooth surface, and this effect on cell wall structure was consistent with that reported for this same serum fraction in an experimental model of disseminated candidiasis in rabbits [31]. Other authors have reported that *C. albicans* exposed to a peptide from chilli pepper seeds caused severe alterations in bud formation, the cell wall and the cytoplasmic membrane of the yeast [42] and the antifungal ceragenins CSA-131 induced *Candida auris* cells to buckle in upon themselves, some of them appearing as merged [43]. Changes in cell wall structure are relevant for viability since they may disturb the traffic of nutrients and the osmotic stability of fungi; in this line, the mAb C7 affected the surface of *C. albicans* blastospores and altered the iron uptake pathway [38, 44]. In the present work we verified that the CAGTA-enr IgG serum fraction of IC patients reduced the metabolic activity of *C. albicans* and induced cell wall modifications, and purified CAGTA eventually killed cells*,* as evidenced by DiBAC staining.

With reference to the antifungal effect of sera from patients infected by NAC species, on average they reduced the metabolic activity or viability of *C. albicans* cells to a lesser extent, comparable to that produced by serum from *C. albicans*-infected patients who presented a low CAGTA titer. The fact that sera from patients with NAC infections may have CAGTA titers, and thus exert some activity against *C. albicans,* could be explained by cross-reactivity, a phenomenon previously described by other authors [13, 27, 39, 45, 46]. In this line, sera from patients infected with *C. parapsilosis*, *C. tropicalis*, *C. glabrata* or *C. dubliniensis* reacted with a recombinant amino terminal section of hyphal wall protein (Hwp1N), a well-characterized cell surface protein expressed only in *C. albicans* hyphae [13, 47]. Similarly, sera from patients infected with *C. tropicalis*, *C. parapsilosis*, *C. glabrata* or *C. guilliermondii* also reacted with a recombinant *C. albicans* enolase-1 (Eno1) [46, 48]. Eno1 is currently considered a moonlighting protein, a glycolysis and gluconeogenesis enzyme that can be secreted to the cell wall of *Candida* spp.[49] and is well known for promoting a strong humoral immune response in patients with invasive candidiasis [50]. Antibodies reacting with methionine synthase protein 6 (Met6), an essential protein for methionine biosynthesis located in the outer layers of *C. albicans* germ tubes cell wall [51] were also found in patients infected not only by *C. albicans* but by NAC species as well [27, 52]. Interestingly, antibodies reacting with Hwp1 and Met6, both proteins targets of CAGTA, resulted good biomarkers of IC in immunocompromised patients, either alone or in combination with beta-D-glucan detection results [27].

Another point to consider with reference to invasive candidiasis is the development of vaccines, and immunization trials in animal models have been conducted with encouraging success rates using various *C. albicans* components such as heat shock protein 90 (Hsp90) [53], Hwp1-N, Eno1[54] and Met6 among others [55, 56]. Some of these proteins react with CAGTAs, and the identification of new targets would provide us with new candidates as possible immunogenic protective agents against invasive candidiasis. Currently, our research group is searching for additional antigens recognized by CAGTA (data not published). So far, only the NDV-3A vaccine (NovaDigm Therapeutics, Inc.) based on Als3p (agglutinin-like sequence protein) is available, and is in phase 3 clinical trial; however, given that this vaccine is intended only for protection against Recurrent Vulvovaginal Candidiasis, the discovery of a vaccine for Invasive Candidiasis remains elusive and requires further investigation.

In conclusion, based on the proposed objective, our study demonstrated that IgG serum fraction from patients with *C. albicans* IC reduces the growth and metabolic activity of planktonic *C. albicans* cells in vitro; in addition, purified CAGTAs modify the surface of hyphal cell walls and, eventually, lead to cell death. Moreover, patients infected with non-*albicans* *Candida* species also produce antibodies that can cross-react with *C. albicans*, and reduce its metabolic activity and viability, although with lower intensity. The findings suggest that CAGTAs and related antibodies could be valuable as diagnostic and prognostic markers for invasive candidiasis, and their target antigens may have potential in the development of immunization protocols against *Candida* infections. Additional investigation is required to validate these discoveries, delve into their clinical implications, and assess the antibodies' capacity to effectively combat the infection.

## References

[1] Kullberg BJ, Arendrup MC (2015). Invasive candidiasis. N Engl J Med.

[2] Kotey FC, Dayie NT, Tetteh-Uarcoo PB, Donkor ES (2021). *Candida* bloodstream infections: changes in epidemiology and increase in drug resistance. Infect Dis Ther.

[3] Bassetti M, Garnacho-Montero J, Calandra T (2017). Intensive care medicine research agenda on invasive fungal infection in critically ill patients. Intensive Care Med.

[4] Pemán J, Zaragoza R (2012). Hacia el diagnóstico temprano de la candidiasis invasora en el paciente crítico. Rev Iberoam Micol.

[5] Strollo S, Lionakis MS, Adjemian J, Steiner CA, Prevots DR (2016). Epidemiology of hospitalizations associated with invasive candidiasis, United States, 2002–2012(1). Emerg Infect Dis.

[6] Lehrnbecher T, Armstrong-James D, Carvalho A (2017). Immunotherapy of invasive fungal disease. Immunogenetics of fungal diseases.

[7] Moragues MD, Rementeria A, Sevilla MJ, Eraso E, Quindos G (2014). *Candida* antigens and immune responses: implications for a vaccine. Expert Rev Vaccines.

[8] Yapar NN (2014). Epidemiology and risk factors for invasive candidiasis. Ther Clin Risk Manag.

[9] Agnelli CC (2022). Prognostic factors of *Candida* spp. bloodstream infection in adults: a nine-year retrospective cohort study across tertiary hospitals in Brazil and Spain. Lancet Reg..

[10] Barantsevich N, Barantsevich E (2022). Diagnosis and treatment of invasive candidiasis. Antibiotics.

[11] Bassetti M, Giacobbe DR, Vena A (2019). Incidence and outcome of invasive candidiasis in intensive care units (ICUs) in Europe: results of the EUCANDICU project. Crit Care.

[12] Garcia-Ruiz JC, del Carmen AM, Regulez P, Quindos G, Alvarez A, Ponton J (1997). Detection of antibodies to *Candida albicans* germ tubes for diagnosis and therapeutic monitoring of invasive candidiasis in patients with hematologic malignancies. J Clin Microbiol.

[13] Moragues MD, Ortiz N, Iruretagoyena JR (2004). Evaluation of a new commercial test (*Candida albicans* IFA IgG) for the serodiagnosis of invasive candidiasis. Enferm Infecc Microbiol Clin.

[14] Parra-Sánchez M, Breval IZ, Méndez CC (2017). *Candida albicans* germ-tube antibody: evaluation of a new automatic assay for diagnosing invasive candidiasis in ICU patients. Mycopathologia.

[15] Pemán J, Zaragoza R, Quindós G (2011). Clinical factors associated with a *Candida albicans* germ tube antibody positive test in intensive care unit patients. BMC Infect Dis.

[16] Pontón José (2006). El diagnóstico microbiológico independiente del cultivo en la candidiasis invasora. importancia de los marcadores fúngicos. Rev Iberoam Micol.

[17] Pontón J, del Palacio A (2007). Avances y limitaciones del diagnóstico precoz de las infecciones invasoras causadas por levaduras. Rev Iberoam Micol.

[18] Quindós G, Pontón J, Cisterna R (1987). Detection of antibodies to *Candida albicans* germ tube in the diagnosis of systemic candidiasis. Eur J Clin Microbiol Infect Dis.

[19] Iruretagoyena JR, Regúlez P, Quindós G, Pontón J (2006). Anticuerpos anti-micelio de *Candida albicans* en dos pacientes de cuidados intensivos con candidiasis invasora. Rev Iberoam Micol.

[20] Zaragoza R, Pemán J, Quindós G (2009). Kinetic patterns of *Candida albicans* germ tube antibody in critically ill patients: Influence on mortality. Clin Vaccine Immunol.

[21] Bujdakova H, Paulovičová E, Borecka-Melkusova S (2008). Antibody response to the 45 kDa *Candida albicans* antigen in an animal model and potential role of the antigen in adherence. J Med Microbiol.

[22] Netea MG, Joosten LA, Latz E (2016). Trained immunity: a program of innate immune memory in health and disease. Science.

[23] Rodier M, Imbert C, Kauffmann-Lacroix C, Daniault G, Jacquemin J (2003). Immunoglobulins G could prevent adherence of *Candida albicans t*o polystyrene and extracellular matrix components. J Med Microbiol.

[24] Shukla M, Chandley P, Rohatgi S (2021). The role of B-cells and antibodies against *Candida* vaccine antigens in invasive candidiasis. Vaccines.

[25] Elluru SR, Kaveri SV, Bayry J (2015). The protective role of immunoglobulins in fungal infections and inflammation. Semin Immunopathol.

[26] Wich M, Greim S, Ferreira-Gomes M (2021). Functionality of the human antibody response to *Candida albicans*. Virulence.

[27] Díez A, Carrano G, Bregón-Villahoz M (2021). Biomarkers for the diagnosis of invasive candidiasis in immunocompetent and immunocompromised patients. Diagn Microbiol Infect Dis.

[28] Matthews RC, Burnie JP (2004). Recombinant antibodies: A natural partner in combinatorial antifungal therapy. Vaccine.

[29] Pitarch A, Nombela C, Gil C (2011). Prediction of the clinical outcome in invasive candidiasis patients based on molecular fingerprints of five anti-*candida* antibodies in serum. Mol Cell Proteom.

[30] Ulrich S, Ebel F (2020). Monoclonal antibodies as tools to combat fungal infections. J Fungi.

[31] Carrano G, Paulone S, Lainz L, Sevilla M, Blasi E, Moragues M (2019). Anti-*Candida albicans* germ tube antibodies reduce in vitro growth and biofilm formation of *C. albicans*. Rev Iberoam Micol.

[32] Ramage G, Vande Walle K, Wickes BL, Lopez-Ribot JL (2001). Standardized method for in vitro antifungal susceptibility testing of *Candida albicans* biofilms. Antimicrob Agents Chemother.

[33] Henriques M, Azeredo J, Oliveira R (2006). *Candida albicans* and *Candida dubliniensis*: comparison of biofilm formation in terms of biomass and activity. Br J Biomed Sci.

[34] Bowman JC, Hicks PS, Kurtz MB (2002). The antifungal echinocandin caspofungin acetate kills growing cells of *Aspergillus fumigatus* in vitro. Antimicrob Agents Chemother.

[35] Fujibayashi T, Nakamura M, Tominaga A (2009). Effects of IgY against *Candida albicans* and *Candida* spp. adherence and biofilm formation. Jpn J Infect Dis.

[36] Machová E, Bystrický S (2008). Growth inhibition of *Candida albicans* in the presence of antiserum elicited in rabbits by mannan-protein conjugate. Z Naturforsch C.

[37] Zhang H, Jia C, Xi H, Li S, Yang L, Wang Y (2011). Specific inhibition of *Candida albicans* growth in vitro by antibodies from experimental *Candida* keratitis mice. Exp Eye Res.

[38] Brena S, Omaetxebarria MJ, Elguezabal N, Cabezas J, Moragues MD, Ponton J (2007). Fungicidal monoclonal antibody C7 binds to *Candida albicans* Als3. Infect Immun.

[39] Antoran A, Aparicio-Fernandez L, Pellon A (2020). The monoclonal antibody Ca37, developed against *Candida albicans* alcohol dehydrogenase, inhibits the yeast in vitro and in vivo. Sci Rep.

[40] Uppuluri P, Singh S, Alqarihi A (2018). Human anti-Als3p antibodies are surrogate markers of NDV-3A vaccine efficacy against recurrent vulvovaginal candidiasis. Front Immunol.

[41] Beucher B, Marot-Leblond A, Billaud-Nail S, Oh S, Hoyer LL, Robert R (2009). Recognition of *Candida albicans* Als3 by the germ tube-specific monoclonal antibody 3D9. 3 FEMS Microbiol Immunol..

[42] Ribeiro SF, Carvalho AO, Da Cunha M (2007). Isolation and characterization of novel peptides from chilli pepper seeds: antimicrobial activities against pathogenic yeasts. Toxicon.

[43] Hashemi MM, Rovig J, Holden BS (2018). Ceragenins are active against drug-resistant *Candida auris* clinical isolates in planktonic and biofilm forms. J Antimicrob Chemother.

[44] Brena S, Cabezas-Olcoz J, Moragues MD (2011). Fungicidal monoclonal antibody C7 interferes with iron acquisition in *Candida albicans*. Antimicrob Agents Chemother.

[45] Pazos C, Moragues M, Quindós G, Pontón J, del Palacio A (2006). Diagnostic potential of (1, 3)-b-D-glucan and anti-*Candida albicans* germ tube antibodies for the diagnosis and therapeutic monitoring of invasive candidiasis in neutropenic adult patients. Rev Iberoam Micol.

[46] Li F, Ma C, Shi L (2013). Diagnostic value of immunoglobulin G antibodies against *Candida* enolase and fructose-bisphosphate aldolase for candidemia. BMC Infect Dis.

[47] Laín A, Elguezabal N, Brena S (2007). Diagnosis of invasive candidiasis by enzyme-linked immunosorbent assay using the N-terminal fragment of *Candida albicans* hyphal wall protein 1. BMC Microbiol.

[48] Clancy CJ, Nguyen ML, Cheng S (2008). Immunoglobulin G responses to a panel of *Candida albicans* antigens as accurate and early markers for the presence of systemic candidiasis. J Clin Microbiol.

[49] Arvizu-Rubio VJ, García-Carnero LC, Mora-Montes HM (2022). Moonlighting proteins in medically relevant fungi. PeerJ.

[50] Strockbine NA, Largen MT, Zweibel SM, Buckley HR (1984). Identification and molecular weight characterization of antigens from *Candida albicans* that are recognized by human sera. Infect Immun.

[51] Saez-Roson A, Sevilla M, Moragues M (2014). Identification of superficial *Candida albicans* germ tube antigens in a rabbit model of disseminated candidiasis. A proteomic approach. Int Microbiol.

[52] Pitarch A, Nombela C, Gil C (2007). Reliability of antibodies to *Candida* methionine synthase for diagnosis, prognosis and risk stratification in systemic candidiasis: a generic strategy for the prototype development phase of proteomic markers. Proteomics Clin Appl.

[53] Raska M, Běláková J, Wudattu NK (2005). Comparison of protective effect of protein and DNA vaccines hsp90 in murine model of systemic candidiasis. Folia Microbio.

[54] Xin H, Dziadek S, Bundle DR, Cutler JE (2008). Synthetic glycopeptide vaccines combining β-mannan and peptide epitopes induce protection against candidiasis. Proc Natl Acad Sci.

[55] Adams AL, Eberle K, Colon JR, Courville E, Xin H (2021). Synthetic conjugate peptide fba-Met6 (MP12) induces complement-mediated resistance against disseminated *Candida albicans*. Vaccine.

[56] Xin H (2019). Effects of immune suppression in murine models of disseminated *Candida glabrata* and *Candida tropicalis* infection and utility of a synthetic peptide vaccine. Med Mycol.

